# Reviewed article: Silva TFPLA, Peixoto HM, Freitas LRS, Araújo ELL, Ramalho WM. Trends in dengue incidence and lethality: interrupted time series analysis, Brazil, 2001-2022. Epidemiol Serv Saude. 2025:34;e20240424.

**DOI:** 10.1590/S2237-96222025v34e20240424.a

**Published:** 2025-09-08

**Authors:** Marilia Mastrocolla de Almeida Cardoso

**Affiliations:** 1 Hospital das Clinicas Botucatu

**Table:** 

Rating	Excellent	Good	Average	Below average	Poor
Interest		✓			
Quality			✓		
Originality		✓			
Overall		✓			

**Table:** 

Questionnaire	Yes	No	Not applicable
Does the manuscript contain new and significant information to justify publication?	✓		
Does the Abstract (Summary) clearly and accurately describe the content of the article?	✓		
Is the problem significant and concisely stated?	✓		
Are the methods described comprehensively?	✓		
Are the interpretations and conclusions justified by the results?	✓		
Is adequate reference made to other work in the field?	✓		

## Manuscript Structure

Length of article is: Adequate

Number of tables is: Too many

Number of figures is: Too few

Please state any conflict(s) of interest that you have in relation to the review of this paper (state “none” if this is not applicable): None

## Comments

O estudo apresenta contribuições para a implementação de políticas para a área de estudo. No entanto, o delineamento do estudo precisa ser mais adequado ao objetivo do estudo. O estudo de fato é um análise descritiva de série temporal, avaliando letalidade e incidência. Para ser caracterizado ecológico necessitaria adicionar mais variáveis que analisam a relação da doença/condição de saúde e a exposição de interesse (exemplo: condições sanitárias, tipos de intervenção) por grupos/regiões/macro regiões).

Com relação às figuras/tabelas, são citadas 2 figuras no texto, mas que estão apresentadas em formato de tabelas. Sugiro corrigir e adequar às orientações da revista. Ainda sobre essa questão, por ser uma série temporal, sugiro incluir de fato figuras ao invés de tabelas para alguns dados, pois facilitam a visualização e distribuição.

Por fim, sugiro rever a discussão. Citações das tabelas/figuras devem ficar restritas ao campo dos resultados. Novos resultados devem ser transferidos da discussão para os resultados. Sugiro rever a organização dos tópicos, de forma a ficar mais didática e de fácil compreensão. Talvez organizar a discussão a partir das variáveis. 

